# Relationships between urbanization and CO_2_ emissions in China: An empirical analysis of population migration

**DOI:** 10.1371/journal.pone.0256335

**Published:** 2021-08-18

**Authors:** Tengfei Zhang, Yang Song, Jun Yang

**Affiliations:** 1 School of Public Finance and Taxation, Southwestern University of Finance and Economics, Chengdu, Sichuan, China; 2 School of Economics and Business Administration, Chongqing University, Chongqing, China; Institute for Advanced Sustainability Studies, GERMANY

## Abstract

China’s announcement of its goal of carbon neutrality has increased the practical significance of research on carbon dioxide (CO_2_) emissions that result from urbanization. With a comprehensive consideration of population migration in China, this study examines the impact of urbanization on CO_2_ emissions based on provincial panel data from 2000 to 2012. Two indicators (resident population and household registration population) are used to measure urbanization rate. The results reveal that the impact of urbanization on CO_2_ emissions in China is closely correlated with the structure of urban resident population and interregional population migration. The estimation results are still robust by using generalized method of moments (GMM) estimator and two-stage least squares (2SLS) estimator. The proportion of temporary residents is introduced as a proxy variable for population migration. The panel threshold model regression results show that the proportion of temporary residents has a marginal effect on the relationship between urbanization and CO_2_ emissions. In regions with a higher proportion of temporary residents, the positive effects of resident population urbanization on CO_2_ emissions tend to be weaker. These findings are consistent with the theories of ecological modernization and urban environmental transition. This paper makes suggestions on China’s urbanization development model and countermeasures are proposed to minimize the CO_2_ emissions caused by urbanization.

## 1. Introduction

With the rapid progress of urbanization, China has also experienced a rocketing growth of carbon dioxide (CO_2_) emissions. China’s urbanization rate increased from 17.9% to 60.6% between 1978 and 2019, with an average annual growth rate of 1%. Moreover, China has become the largest emitter of CO_2_ in the world. China’s CO_2_ emissions accounted for 28.5% of the world’s total carbon emissions in 2014, whereas its proportion of world emissions was only 13.7% in 2000 [1]. Carbon emission reduction affects China’s sustainable economic and environmental development. In September 2020, the Chinese government announced its efforts to aim for a peak in CO_2_ emissions by 2030 and carbon neutrality by 2060. In addition, China expressed its intention to formulate an action plan that achieves the peak of CO_2_ emissions before 2030. Increasing attention to climate change policies and energy policies has stimulated an examination of the relationship between urbanization and CO_2_ emissions in China.

Scholars have yet to obtain consistent results from research on the relationship between urbanization and CO_2_ emissions. A number of scholars have argued that this relationship is non-linear [2–4], while others have found that the relationship may be in fact linear [5, 6]. The inconsistency in the existing literature can be put down to differences in research samples and empirical methods. In all likelihood, the characteristics of population migration could also be one of these factors in China.

The current statistical calibration of China’s urban population includes the country’s temporary residents who live in urban areas longer than half a year, most of them are rural migrant workers who migrate to cities from rural areas for seeking temporary employment and opportunities of higher incomes. According to the 2020 China’s Statistical Bulletin on National Economic and Social Development, the total number of China’s rural migrant workers is 285.6 million, which takes up 20.23% of China’s total population. The relationship between urbanization and CO_2_ emissions in China may be closely associated with the unique characteristics of migration and the different stages of urbanization across regions.

Due to the constraint of the household registration (hukou) system, the migration population in China’s urbanization process has individualized features. The hukou system has divided China’s population into urban and rural citizens. Defined as household registration, hukou is a special way of population management in China. It records the basic information of an individual citizen, such as name, date of birth, kinship, legal address, etc. As such, it is difficult for rural residents to obtain urban hukou in cities, limiting their access to corresponding urban welfare and social benefits, such as high-quality education, medical care, and other public services. In the forty years following the implementation of the policy of "Reform and Opening-Up", a sizeable population of rural migrants have moved to urban areas. This migrating population has a complex social structure, with a proportion of migrants that has obtained an urban hukou and thus become members of the urban population. The remaining proportion of migrants, however, have been unable to obtain an urban hukou despite living in cities and thus remain registered under the rural hukou. Rural migrant workers, who possess rural hukou, are a unique example of this generally highly mobile population group. The net population inflow thus tends to be positive in areas with a more-developed local economy, which will change the characteristics of urban population structural. Cities in the stage of highly developed urbanization have a greater attraction to migration population. As urban population agglomerates, the environmental problems related to (water and air) pollution and (energy and carbon emission) consumption will emerge [7]. Considering this population migration, the influence of urbanization on CO_2_ emissions across China’s various provinces (municipalities and autonomous regions) is worthy of investigation, as the topic has practical significance for green and sustainable urban development in China.

This study adds to current literature with three aspects. First, previous studies tended to only include a single indicator in their measurement of urbanization without considering the complex structures of urban population growth in China. This omission is likely to lead to systematic estimation errors. Temporary urban residents are a standard feature of China’s urbanization process. In order to acquire an in-depth understanding of the impact of temporary residents on CO_2_ emissions, this study introduces two types of urbanization indicators. Secondly, the combination of urbanization, population migration and carbon emissions, which examines the marginal impact of temporary resident population on the relationship between urbanization and carbon emissions, provides a theoretical foundation for the policy making of urban carbon emission reduction and urban population growth in developing economies. Thirdly, this paper uses a series of historical variables and economic variables as instrumental variables and amplifies the solutions to the endogeneity problem of urbanization rate.

The goals of this study are to explore the impact of urbanization on CO_2_ emissions in China with a comprehensive consideration of population migration, to analyze the reasons for various impacts of different urbanization rates on carbon emissions, and to propose suggestions for a more sustainable urbanization process. Panel data covering thirty provinces (municipalities and autonomous regions) is used for the analysis. The results show that the impact of urbanization on CO_2_ emissions is closely correlated to urban population structure. Results are still robust after using different estimation models and solving the endogenous problem. The proportion of temporary residents has a significant marginal effect on the relationship between urbanization and CO_2_ emissions. The study is structured as follows: Section 2 presents the literature review; Section 3 introduces the models and resources used; Section 4 provides statistical descriptions of China’s inter-provincial migration patterns; Section 5 presents the results and discussion; and the final section includes the conclusions and suggested countermeasures.

## 2. Literature review

Urbanization is an important factor in China’s economic development, influencing urban economic growth through industry distribution, land consumption, human capital, and government expenditure [8–11]. The emissions of CO_2_ that are generated by the urbanization process have drawn greater academic attention. Chen et al. [6] explored urbanization from the dimensions of population size, land area, and economic strength to verify the positive impact of urbanization on CO_2_ emissions in China. Lv et al. [5] found that urbanization had a significant positive effect on CO_2_ emissions due to its increases in road and air transportation; however, the CO_2_ emissions from railway and water transportation were significantly negatively correlated with urbanization. Some studies have emphasized that urbanization has a non-linear impact on CO_2_ emissions, demonstrating an undulating threshold effect [3, 12]. In addition, per-capita CO_2_ emissions that result from urbanization deserves greater attention. Coordination between urbanization and low-carbon development appears to be closely associated to the urban development stage and geographic location [13]. Zhang et al. [14] measured the center of gravity trajectory of CO_2_ emissions and found that the center of gravity shifted to Northwest to a greater extent throughout the sample period. A number of studies have focused on the efficiency of CO_2_ emissions in urbanization. Liu et al. [15] believed that the demographic and economic effects of mature urban agglomerations improved the efficiency of CO_2_ emissions. Li et al. [16] identified a U-shaped relationship between urbanization and CO_2_ emission efficiency in cities along the Yangtze River Delta. However, Zhou et al. [17] found that carbon emission performance and urbanization showed a positive spatial dependence among China’s 30 provinces.

The mechanisms behind the impact of urbanization on CO_2_ emissions are also a matter of concern. Ma [18] suggested that the long-term net effects of urbanization on energy and power intensity are significantly positive, while infrastructure construction was the most significant channel of influence. It has also been found that urbanization’s contribution to CO_2_ emissions stems from changes in urban household energy use and the transportation and commercial sectors, the impact of which differs significantly between regions [19]. The power, heat and transportation sectors were the most-consuming sectors associated with the increase in CO_2_ emissions in urbanization [20]. The increase in CO_2_ emissions of urban households is attributed to the increase in the consumption of high-energy consumer electronics, driven by the rise in urban residents’ income and expenditure [21]. Using Shandong Province as the location for a case study, Ren et al. [22] determined that the province’s coal and oil energy structure accelerated the CO_2_ emissions associated with its urbanization. Hence, when adopting a sustainable urban planning system, the government should attempt to strike a balance between urbanization and sustainability [23].

It was also found that the impact of urbanization on CO_2_ emissions differs significantly across regions and development stages, while the main driving factors include technological constraints, wealth and population [24, 25]. Cui et al. [26] constructed a comprehensive coordinated development index to assess the sustainability of urbanization in the Beijing-Tianjin-Hebei region and found that the correlation between urbanization efficiency and environmental quality increased during the sample period. In a case study of Guangdong Province, Wang et al. [27] explored the coupling of energy efficiency and urbanization, which finds that only Guangzhou and Shenzhen were in a high-coupling stage, while other cities in the province were undergoing low- and medium-coupling stages. Yang et al. [28] found a significant correlation between the urbanization level and ecosystem service value in the urban agglomeration in Central Yunnan (China). Other studies have found that land and economic urbanization in the Pearl River Basin positively affected CO_2_ emissions, while population urbanization reduced CO_2_ emissions, and land use, industry production, output accumulation, and energy consumption patterns were the most significant influential factors [29, 30].

The existing studies have deepened the understanding of the relationship between urbanization and CO_2_ emissions, exploring the mechanisms behind such impacts and how they differ by region and industry. There are also other literatures of the economic, welfare and health effects of migration in the process of urbanization [31–33]. Todaro [34] revealed the impact of rural-urban labor migration on urban unemployment and underemployment using a behavioral model. Zhu et al. [35] found return migrants play a positive role in promoting local economic development and rural-urban transformation. China’s large cities and urban agglomerations are the most attractive place for rural migrants where medical amenities, educational amenities and transportation services exert a significantly positive effect on rural-urban migrants’ intentions [36–38]. Rural migrants earn considerable income and make contribution to the sustainable growth of urban labor productivity in Chinese cities [39]. Due to the hukou system, the migrant population in cities has a significant impact on China’s urbanization process [40, 41]. This study intends to construct distinct urbanization indicators to empirically investigate the impact of urbanization on CO_2_ emissions in various regions. In addition, the proportion of temporary residents is used as a proxy and threshold variable for population migration, and the panel threshold model is used to test the marginal effects of migration on the relationship between urbanization and CO_2_ emissions. Based on conclusions of the study, policy suggestions are proposed regarding sustainable development and urban population development strategies.

## 3. Model and materials

### 3.1. Empirical model

In this section, we employ the equation of "Impact = Population×Affluence×Technology (IPAT)", which has been proposed by Ehrlich and Holdren [42], to explore the relationship between urbanization and CO_2_ emissions. The IPAT equation refers to the impact of economic activity on the environment, in which *I* denotes the environment, *P* denotes the population size, *A* denotes affluence or per capita consumption (usually proxied in terms of gross domestic product (GDP) per capita), and *T* is the technological factor. However, the IPAT equation has two drawbacks. First, it is only a mathematical equation and is inappropriate for testing hypotheses. Second, it assumes a rigid elasticity of environmental impact to population, affluence and technology. In response, Dietz and Rosa [43] have developed a stochastic version of IPAT, that is known as the Stochastic Impacts by Regression on Population, Affluence and Technology (STIRPAT) model. The model is given by:

$$I_{it}=\alpha_{i}P_{it}^{\beta }A_{it}^{\gamma }T_{it}^{\delta }\varepsilon_{it}$$ (1)

where the subscript *i* denotes the unit of analysis and *t* denotes the time period. *α*_*i*_ is the constant term and *ε*_*it*_ represents the random error term. When all variables are expressed in natural logarithms, the model can be written as follows:

$$\ln(I_{it})=\alpha +\beta \ln(P_{it})+\gamma \ln(A_{it})+\delta \ln(T_{it})+\varepsilon_{it}$$ (2)

To investigate the effects of urbanization on CO_2_ emissions, the STIRPAT model can be easily rearranged with additional explanatory variables. The augmented model can be interpreted as follows:

$$\begin{array}{l} \ln(CO_{2,it})=\alpha_{0}+\beta_{1}\ln(POP_{it})+\beta_{2}\ln(GDP_{it})+\beta_{3}\ln(HEAVY_{it})+\beta_{4}\ln(URB_{it})+\beta_{5}\ln(X_{it})\\ \;+year_{t}+id_{i}+\varepsilon_{it} \end{array}$$ (3)

Here, *POP* is measured by population density, *GDP* is proxied by GDP per capita and *HEAVY* is measured by the share of heavy industry output in gross value of industrial output. *URB* denotes the urbanization variables and *X* is the set of other explanatory variables, including foreign direct investment (*FDI*), environmental regulation and energy consumption. *year*_*t*_ and *id*_*i*_ denotes the time fixed effects and regional fixed effects, respectively.

As in the analysis for model (3), we consider the fixed effects model (FE). The fixed effects model with Driscoll-Kraay standard errors (DK) is also employed because it can solve heteroscedasticity and autocorrelation problems; the standard error estimates are robust to general forms of cross-sectional and temporal dependence [44]. The Hausman test is performed to check whether the FE estimate is appropriate.

Remarkably, this analysis does not account for the implicit correlation of the dependent variable in model (3), i.e., CO_2_ emissions in the current period have a pronounced impact on the lagged CO_2_ emissions. To overcome the limitations of the static panel estimation techniques, we employ generalized method of moments (GMM) estimator, which allows us to more effectively managed problems such as joint endogeneity of the independent variables and heterogeneity [45, 46]. The dynamic version of model (3) can be written as follows:

$$\begin{array}{l} \ln(CO_{2,it})=\alpha_{0}+\varphi \ln CO_{2,i,t-1}+\beta_{1}\ln(POP_{it})+\beta_{2}\ln(GDP_{it})+\beta_{3}\ln(HEAVY_{it})+\beta_{4}\ln(URB_{it})\\ \;\beta_{5}\ln(X_{it})+\varepsilon_{it} \end{array}$$ (4)

where *CO*_*2*,*i*,*t-1*_ represents the lagged term of dependent variable. The ordinary least squares (OLS) and static panel estimation techniques exhibit large biases compared to the GMM estimators [45]. In this context, we use the first-differenced GMM estimators and the system GMM estimators to estimate the impact of urbanization on CO_2_ emissions across China’s provinces (municipalities and autonomous regions). However, the first-differenced GMM estimators exhibit a large finite sample bias when the autoregressive parameter is moderately large and the number of time series observations is moderately small [47, 48]. In this case, the instruments available for the first-difference equation are weak. Therefore, the system GMM estimator is employed as the primary estimation technique to estimate model (4). Given that the standard error of the two-step estimators might be severely biased downwards, resulting in mechanically low p-values, the finite sample corrected standard errors is implemented [49].

To investigate the role of population migration in explaining the relationship between urbanization and CO_2_ emissions, we build a panel threshold model [50]. The equation is as follows:

$$\begin{array}{l} \ln(CO_{2,it})=\alpha_{0}+\beta_{1}\ln(POP_{it})+\beta_{2}\ln(GDP_{it})+\beta_{3}\ln(HEAVY_{it})+\lambda_{1}\ln(URB_{it})I(TEM_{it}\leq \phi_{1})\\ \;+\lambda_{2}\ln(URB_{it})I(TEM_{it}>\phi_{1})+\beta_{4}\ln(X_{it})+\varepsilon_{it} \end{array}$$ (5)

where I(·) is the indicator function. *TEM*_*it*_ represents provincial proportion of temporary residents, a proxy for population migration in China, is the threshold variable. *φ*_*1*_ is the optimal threshold value of population migration. The two regimes are distinguished by differing regression slopes *λ*_*1*_ and *λ*_*2*_.

### 3.2. Data

In this study, the proportion of temporary residents to total residents in each region is introduced as a proxy variable for population migration. Temporary residents data is extracted from the China Temporary Resident Population Statistical Yearbook issued by the Ministry of Public Security of China. In the yearbooks from 2001 to 2012, temporary residents are divided into three groups based on the duration of their stay (less than 1 month, from 1 month to 1 year, and more than 1 year). Temporary residents that stay in cities for less than 1 month are excluded from the analysis in this study. The total number of temporary residents over one year in a given city is calculated by adding half of the number of those who stay between 1 month to 1 year with the number of those who have stayed for more than 1 year. Resident population refers to the population who lives in a certain area, while in China, it refers to the population who actually lives in a certain area for more than half a year. As such, only temporary population living in a city for over half a year and resident population are included in the calculations. The ratio of temporary residents to resident population has certain economic implications. Changes in the proportion of temporary residents affect regional welfare, resource allocation, environmental protection, and resource development and protection via labor, employment and distribution ratio of wealth accumulation and consumption, as well as the distribution and scale of the market. In the China Temporary Resident Population Statistical Yearbook released after 2013, the standard for inclusion in the category of temporary residents is changed to less than half a year, half a year to 5 years, and more than 5 years. This change in statistical standard is significant; thus, in order to maintain consistency in the data, only samples from 2001 to 2012 are included in the regression analysis.

Our estimations are conducted using panel data across thirty provinces (municipalities and autonomous regions) in China during the period 2000–2012, excluding Tibet, Hong Kong, Macau and Taiwan because of data limitations. The dependent variable is China’s CO_2_ emissions, which are estimated within the framework of the IPCC due to a lack of official statistics. Taking into account China’s hukou system, the index of urbanization is subdivided into two indicators: the urbanization rate of resident population and the urbanization rate of household registration population. The urbanization rate of resident population refers to the proportion of urban resident population, accounting for both the urban household registration population and rural-urban migration. The urbanization rate of household registration refers to the proportion of urban household registration population. These two urbanization indicators thoroughly consider the evolutionary process of urbanization, we can examine the role of population migration in explaining the effect of urbanization on CO_2_ emissions in China.

Other variables include the following:1) Population density, expressed as the resident population per square kilometre, is a measure of the effect of population size on CO_2_ emissions; 2) GDP per capita, which captures the impact of regional economic development on CO_2_ emissions; 3) Proportion of heavy industry output value, which is measured by the share of heavy industry output value in the gross value of industrial output, thus reflecting the effect of industrial structure on CO_2_ emissions; 4) *FDI*, a measure of the effect of FDI on CO_2_ emissions; 5) Environmental regulation, a measure of the effect of environmental regulation intensity on CO_2_ emissions, defined as investment completed in the treatment of industrial pollution across China’s regions; 6) Energy consumption, which is a measure of the effect of energy consumption on CO_2_ emissions, expressed as the ratio of total energy consumption to GDP across China’s regions; and 7) The number of national high-tech industrial development zones, an instrumental variable of urbanization rate, whose data are collected from 2018 China Development Zone Audit Announcement List issued by China’s National Development and Reform Commission.

The data for the provincial demographics are collected from the China Statistical Yearbook, the China Compendium of Statistics and the Provincial Statistical Yearbook. The remaining data are provided by the China Statistical Yearbook, the China Industry Economy Statistical Yearbook and the China Economic Census Yearbook. Table 1 presents the summary statistics for all of the variables (S1 File).

**Table 1:** Descriptive statistics for all variables.

Variables	Definition	Mean	Median	Max.	Min.	Std. dev.	Obs
*CO*_*2*_	CO_2_ emissions (100 millions tons)	2.168	1.686	9.237	0.09	1.674	390
*RESIDENT*	Urbanization rate of resident population (Percent)	46.669	44.023	89.318	23.2	14.945	390
*HOUSEHOLD*	Urbanization rate of household registration population (Percent)	35.387	31.317	89.76	14.46	16.187	390
*TEM*	Proportion of temporary residents (Percent)	4.785	2.171	32.064	0.306	6.251	390
*DENSITY*	Population density (100 millions persons/10^4^ km^2^)	0.041	0.027	0.375	0.001	0.057	390
*GDP*	GDP per capita (10^4^ Yuan)	2.2	1.64	9.317	0.276	1.727	390
*HEAVY*	Proportion of heavy industry output value (Percent)	69.565	70.265	95.425	34.233	12.676	390
*FDI*	Foreign direct investment (Percent)	90.388	28.557	1159.755	5.427	173.323	390
*ER*	Environmental regulation (100 millions Yuan)	12.525	9.4	84.416	0.101	12.036	390
*EC*	Energy consumption (10^4^ tons SCE/100 millions Yuan)	1.436	1.219	5.834	0.402	0.78	390
*DEVELZONE*	The number of national high-tech industrial development zones	2.077	2	9	0	1.649	390

## 4. Provincial population migration in China

### 4.1. CO_2_ emissions per unit GDP versus urbanization rate of resident population

**Fig 1:**
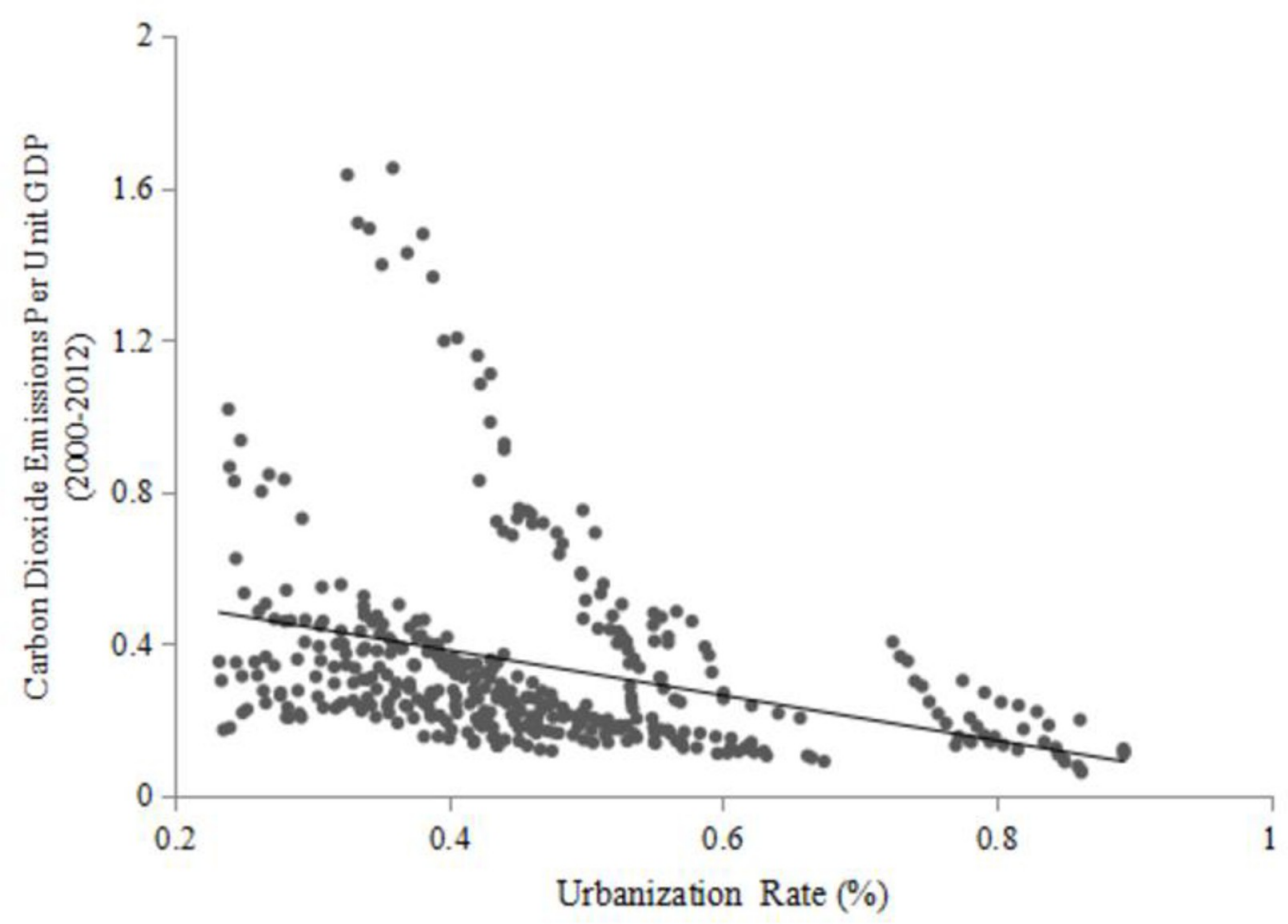
Plot of CO_2_ emissions per unit GDP versus urbanization rate for 2000–2012.

Fig 1 depicts the relationship between CO_2_ emissions per unit GDP and the urbanization rate of resident population across China’s provinces (municipalities and autonomous regions) from 2000–2012 (S1 Fig). The scatter plot shows a strongly significant negative correlation (p<0.0000): the correlation coefficient is -0.342. The scatter plot suggests different behaviors for different provinces (municipalities and autonomous regions) in China: for developing regions such as Shanxi, Inner Mongolia and Ningxia, there appear to be higher values of the pollutant emissions, corresponding to lower values for the urbanization rate. For developed regions such as Beijing, Shanghai and Tianjin, however, there appear to be lower pollutant emissions but a higher urbanization rate. This suggests that China’s economic growth mode not only is shifting from resource intensive to resource saving across regions, but is also accompanied by an increased urbanization rate.

### 4.2. Provincial population migration versus urbanization rate of resident population

**Fig 2:**
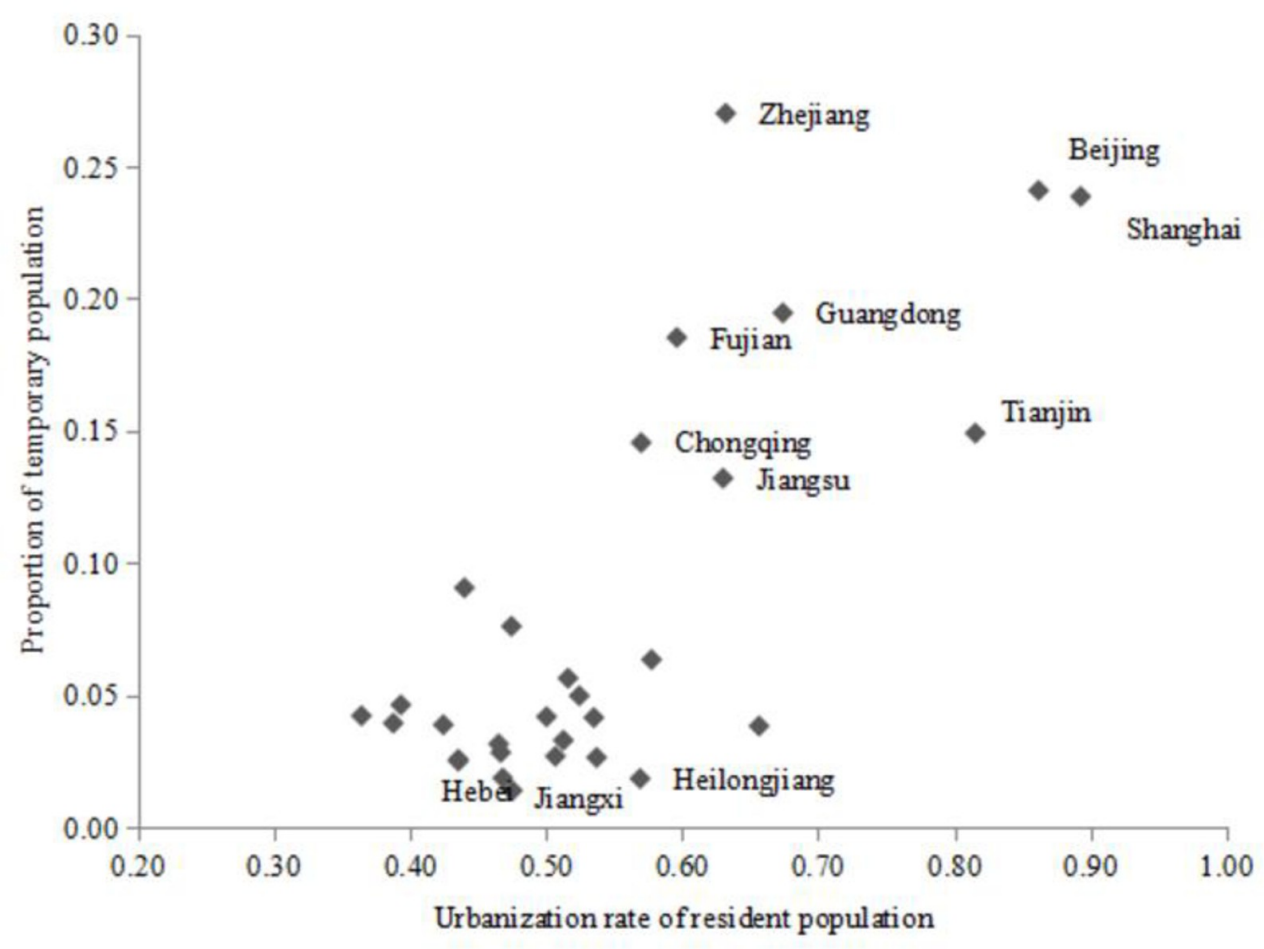
Plot of urbanization rate of resident population versus proportion of temporary residents.

Fig 2 presents a scatter plot of the relationship between the proportion of temporary population and the urbanization rate of resident population across regions in 2012 (S2 Fig). Only temporary and resident population that has remained in the city for more than half a year is included in the analysis. Regions with a high resident population urbanization rate have a high proportion of temporary residents. In the 8 provinces or municipalities in which the proportion of temporary residents is greater than 10%, the urbanization rate of resident population is above 55%. Moreover, 7 of these 8 regions are located in Eastern China. The proportion of temporary residents in the regions of Eastern China is thus significantly higher than that of those in Central and Western China. Eastern China is consequently deemed to be the main region of net inflow for the migrating population. As a comparison, other provinces, such as Hebei, Jiangxi, and Heilongjiang, have a relatively low proportion of temporary residents. Furthermore, inter-provincial migration is found to have an effect on the urbanization process. Therefore, when studying the relationship between urbanization and CO_2_ emissions, factors such as differences in urbanization stages across regions and migration characteristics should be considered.

## 5. Results and analysis

### 5.1. Results from FE and DK estimations

This paper has performed unit root and cointegration tests on panel data before the benchmark regression. The first-order differences of all variables are stationary, and cointegration exists among all variables. Table 2 reports the results of the effects of urbanization on CO_2_ emissions from FE and DK estimations. The results of the Hausman test suggest that the fixed effects model is preferred at the 1% significance level. The estimation of FE model (columns (A) and (B)) has controlled the time-fixed effects and region-fixed effects. Obviously, the results from DK estimations show a more desirable estimation performance than the results from FE estimations. The effects of urbanization on CO_2_ emissions varies widely with different urbanization indices. The estimated coefficient for the urbanization rate of resident population is significantly positive, which is opposite of the estimated coefficient for the urbanization rate of household registration population, the latter coefficient is both negative and statistically significant.

**Table 2:** Urbanization and CO_2_ emissions: FE estimators, DK estimators and 2SLS estimators.

Variables	FE model		DK model		2SLS	
	(A)	(B)	(C)	(D)	(E)	(F)
*RESIDENT*	0.192***		0.152**		1.071***	
	(2.73)		(2.64)		(5.12)	
*HOUSEHOLD*		-0.385**		-0.151***		-0.883*
		(-2.01)		(-3.88)		(-1.76)
*DENSITY*	-0.004	-0.003	-0.003	-0.002	0.001	-0.001
	(-1.1)	(-0.93)	(-0.72)	(-0.76)	(0.09)	(-0.15)
*GDP*	1.062***	1.072***	0.803***	0.872***	0.027**	1.223***
	(19.45)	(19.63)	(26.64)	(29.56)	(2.03)	(9.16)
*HEAVY*	0.17**	0.207***	0.101***	0.133***	0.025	0.245***
	(2.39)	(2.92)	(3.45)	(4.26)	(0.28)	(3.16)
*FDI*	-0.001	-0.002	-0.002	-0.002	-0.008	0.001
	(-0.04)	(-0.1)	(-0.69)	(-0.43)	(-0.92)	(0.14)
*ER*	0.023**	0.028***	0.031***	0.034***	0.07***	0.025**
	(2.16)	(2.63)	(4.56)	(4.75)	(4.7)	(2.13)
*EC*	0.643***	0.689***	0.682***	0.736***	0.555***	0.952***
	(13.18)	(14.18)	(10.71)	(10.88)	(9.86)	(8.13)
Constant	-1.462***	-0.845***	-0.912***	-0.18	-2.44***	0.922
	(-5.59)	(-4.55)	(-3.3)	(-0.67)	(-3.18)	(0.83)
Within R-squared	0.952	0.951	0.946	0.946	0.88	0.927
Hausman test statistics	37.19***	26.95***				
Sargan-Hansen statistics					1.301	3.292
					[0.522]	[0.193]
Observations	390	390	390	390	390	390

Notes: FE denotes fixed effects model; DK denotes fixed effects model with Driscoll-Kraay standard errors. *t*-values are given in parentheses. *p*-values are given in square brackets.

"***", "**" and "*" denote the significance levels at 1%, 5% and 10%, respectively.

Considering that carbon emissions will also affect the urbanization process across China, besides the possibility of measurement errors and missing variables, the model has a potential endogeneity problem. Historical characteristic variables exert an impact on urbanization without directly influencing carbon emissions during the sample period, which thus meets the correlation and exogeneity principles of instrumental variables. This paper selects 1) whether railway went into service in each province in 1937, 2) the provincial railway mileage in operation in 1990 and 3) the provincial population density in 1990 as the instrumental variables of urbanization rate. Meanwhile, the number of national high-tech industrial development zones in each province (municipality or autonomous region) is a driving force of economic development. In addition to being important for the development of urbanization, it has no direct correlation with the explained variables. Hence, this paper also selects the number of national high-tech industrial development zones in each province (municipality or autonomous region) during the period of the sample as the instrumental variable of urbanization. Using the two-stage least squares (2SLS) estimation, the columns (E) and (F) of Table 2 in this paper present the regression results of instrumental variables. There is still a significantly positive relationship between the urbanization rate of resident population and carbon emissions, as well as a significantly negative relationship between the urbanization rate of household registration population and carbon emissions. This reveals that the estimation results of the fixed-effect models are robust.

### 5.2. Results from first-differenced GMM and system GMM estimations

Table 3 reports the results of the effects of urbanization on CO_2_ emissions according to both first-differenced GMM estimations and system GMM estimations. All of the regressions pass the Sargan test, whose null hypothesis is that over-identifying restrictions are valid. Moreover, all of the regressions pass the AR(2) test, whose null hypothesis is that there is no second-order serial correlation in first-differenced errors, thus suggesting that the two-step system GMM estimators are more efficient. It should be noted, however, that the finite sample’s corrected standard errors are implemented in both the first-differenced GMM estimators and the system GMM estimators [49]. Comparing the estimated results in Table 3 with those in Table 2, we find that the significant level of all of the variables declines (more or less); however, the results in Table 3 remain more robust. The coefficient for the urbanization rate of resident population remains both positive and statistically significant at the 1% significance level in the first-differenced GMM estimators, but it becomes statistically insignificant in the system GMM estimators. Likewise, the coefficient for the urbanization rate of household registration population is still significantly negative in the system GMM estimators but becomes statistically insignificant in the first-differenced GMM estimators. The empirical results appear more complicated because their findings vary with different urbanization variables. These findings imply that the effect of urbanization on CO_2_ emissions is closely related to the structure of the urban resident population and the characteristics of population migration in China.

**Table 3:** Urbanization and CO_2_ emissions: First-differenced GMM estimators and system GMM estimators.

Variables	First-differenced GMM model		System GMM model	
	(A)	(B)	(C)	(D)
Lag of dependent variable	0.153*	0.172*	0.555***	0.504***
	(1.69)	(1.76)	(4.6)	(4.22)
*RESIDENT*	0.313***		-0.179	
	(3.94)		(-1.19)	
*HOUSEHOLD*		0.093		-0.161*
		(0.96)		(-1.77)
*DENSITY*	0.002	0.002	0.001	0.001
	(0.76)	(0.95)	(0.03)	(0.21)
*GDP*	0.616***	0.646***	0.455***	0.489***
	(7.63)	(7.38)	(3.78)	(3.85)
*HEAVY*	0.047	0.048	0.07	0.078
	(1.01)	(1.14)	(1.1)	(1.33)
*FDI*	-0.005	-0.005	-0.007*	-0.006
	(-1.33)	(-1.37)	(-1.88)	(-1.56)
*ER*	0.017	0.02*	0.01	0.011
	(1.19)	(1.72)	(1.08)	(1.17)
*EC*	0.51***	0.489***	0.414***	0.459***
	(2.86)	(3.23)	(2.72)	(2.86)
Constant	-1.059	-0.315	0.018	-0.137
	(-1.19)	(-0.43)	(0.03)	(-0.25)
Sargan test	26.541	25.549	25.337	25.205
	[0.149]	[0.181]	[0.233]	[0.238]
AR(2) value	0.961	1.129	1.172	1.183
	[0.337]	[0.259]	[0.241]	[0.237]
Observations	330	330	360	360

Notes: The value in parentheses are the *z*-values, whereas the values in square brackets are the *p*-values.

"***", "**" and "*" denote the significance levels at 1%, 5% and 10%, respectively.

Looking at the other variables, the estimated coefficients on population density are statistically insignificant in all of the models. The estimated coefficients on GDP per capita are both positive and statistically significant at the 1% level in all of the models, which indicates that China’s economic growth is accompanied by the development of high-polluting, energy-intensive industries. The estimated coefficients on the proportion of heavy industry output value are significantly positive in both the FE model and the DK model, but the coefficients become insignificant in the first-differenced model and the system GMM model. The estimated coefficients on FDI are both negative and statistically significant in the system GMM model, rejecting the pollution haven hypothesis, which argues that polluting industries tend to shift from countries with stringent regulations to countries with lax regulations. The estimated coefficients on environmental regulation are statistically significant in the FE and the DK models, and the coefficients become insignificant in the first-differenced and the system GMM models. The estimated coefficients on energy consumption are both positive and statistically significant at the 5% level or lower in all models, which indicates that CO_2_ emissions are closely related to energy consumption in China.

### 5.3. Analysis of the effect of the urbanization rate of resident population on CO_2_ emissions

Remarkably, the estimated coefficients for the urbanization rate of resident population in the FE model, the DK model and the first-differenced GMM model are all significantly positive at the 5% level or lower, thus indicating that urbanization of resident population increases CO_2_ emissions across China’s regions. To gain a deeper understanding of the positive relationship between urbanization of resident population and CO_2_ emissions in China, it is essential to understand both the structure of China’s urban resident population and the characteristics of China’s population migration. The urban resident population can be divided into two categories: 1) the urban household registration population; and 2) rural migration workers. The rural migration workers do not have access to urban public services such as education and health. Additionally, because house prices are sharply increasing, it is more difficult for rural migration workers to purchase homes. These constraints have a strong impact on the lifestyle of rural migration workers, leading to a high frequency of population migration between urban and rural areas. According to the National Bureau of Statistics of China [51], there are 290.77 million rural migration workers in 2019, accounting for 52.71% of the total rural population. For many years, large-scale migration flows have occurred during China’s Spring Festival period. The urban household registration population can enjoy comprehensive public education and health services. They are more likely to consume private vehicles and other energy-intensive products that put additional stress on the environment. According to the data from the Beijing Statistical Yearbook, the number of private vehicle in Beijing has increased from 0.811 million in 2002 to 4.075 million in 2012, while the city’s growth rate from 2002–2012 has averaged 17.8%.

Having a preliminary understanding of the characteristics of China’s urban resident population, it is not difficult to understand the positive correlation between urbanization of resident population and CO_2_ emissions. First, a large share of the population, including rural migrants, congregates in cities, thus substantially increasing urban governance costs and causing higher levels of CO_2_ emissions. Second, with increased income levels, demands for private vehicles and other energy-intensive products increase, resulting in the emission of higher levels of CO_2_. Third, rural migrants’ lifestyles shift from rural to urban, potentially increasing the demand for other goods and services and resulting in higher CO_2_ emissions. It is noted that rural migrants’ activities are not the only factor leading to more CO_2_ emissions. In addition, the construction and operation of the public infrastructure, such as road networks, garbage disposal, sewage systems and electricity networks, may lead to additional CO_2_ emissions.

### 5.4. Analysis of the effect of the urbanization rate of household registration population on CO_2_ emissions

In contrast to the resident population’s urbanization rate, the estimated coefficients for the urbanization rate of household registration population in the FE model, the DK model and the system GMM model are all significantly negative at the 10% level or lower, indicating that the urbanization of household registration population is conducive to carbon emission reduction. Likewise, it is essential to understand the household registration admittance system that is prevalent in China’s cities. Many of China’s cities have established a series of strict household registration admittance systems. If one wants to obtain access to urban household registration, he or she must satisfy a series of harsh conditions, which primarily reflect educational background, professional skills and social insurance contributions. For example, the qualifications to apply for Beijing’s household registration population are only open to specific persons, such as graduates who have found jobs in Beijing, high-calibre personnel and outstanding businessman. Another example is Shanghai: graduates from other provinces (municipalities and autonomous regions) who want to enter Shanghai’s household registration must pass a strict assessment based on a scoring system whose primary elements include educational background, foreign language aptitude and innovation capability.

Currently, it is easy to comprehend the negative relationship between urbanization of household registration population and CO_2_ emissions by using the theory of urban environmental transition. On the one hand, many of China’s cities strictly limit the size of the urban household registration population; one important purpose of this limitation is to prevent the pollution caused by an excessive expanded population. On the other hand, as explained by the theory of urban environmental transition, cities often become wealthier and that wealth is accompanied by manufacturing activities, leading to industrial pollution issues such as air and water pollution [7]. In addition, wealthier cities are often accompanied by consumption-related environmental problems such as vehicle-exhaust pollution and energy consumption. However, the aforementioned issues can be diminished through environmental regulations, technological progress and industrial restructuring. Therefore, as income levels and urban development stages increase, there seems to be a negative correlation between the urbanization of household registration population and CO_2_ emissions.

### 5.5. Results and analysis from threshold regression estimations

The threshold model captures the impact of urbanization on CO_2_ emissions based on population migration (proportion of temporary population). Using Hansen’s [50] threshold model, the *F*-statistics reported in Table 4 clearly reject the null hypothesis of no threshold effect, implying the existence of significant single threshold effect. The estimated threshold value of the population migration index is 0.199 with a 95% confidence interval ranging from [0.197, 0.206] by using urbanization rate of resident population, and also with a 95% confidence interval ranging from [0.197, 0.202] by using urbanization rate of household registration population. When population migration, proxied by the proportion of temporary residents, is below the threshold value, CO_2_ emissions is positively influenced by the urbanization rate of resident population (column (A) of panel B in Table 4). The estimated coefficient is 0.39, which is significant at the level of 5%. Meanwhile, in case population migration index goes beyond the threshold value, the estimated coefficient becomes 0.341, which is statistically significant at the level of 10%, and indicates the positive effect turns to be weak. When population migration, proxied by the proportion of temporary residents, is below the threshold value, the CO_2_ emissions is positively influenced by urbanization rate of household registration population (column (C) of panel B in Table 4). The estimated coefficient is 0.291, which is significant at the level of 5%. However, when population migration index rises, the effect turns to be weak and statistically insignificant. The proportion of temporary residents is introduced as a proxy variable for population migration, as the variable represents the proportion of the population that changes their location of residence spatially and regionally while their hukou status remains unchanged. Based on the data from the collected samples, the temporary residents in a given area do not spatially overlap with the local population that has an urban hukou. To some extent, this finding explains why the significance of the estimated coefficient for the urban hukou urbanization rate in columns (C) and (D) is weaker than the estimated coefficient for the urban resident population in columns (A) and (B) of panel B in Table 4.

**Table 4:** Threshold regression estimation results.

Panel A: Test of threshold effects				
	Single threshold:	Single threshold:	Double threshold:	Double threshold:
	Estimate (95% confidence interval)	F (10%, 5%, 1% critical values)	Estimate (95% confidence interval)	F (10%, 5%, 1% critical values)
*RESIDENT*	0.199** (0.197, 0.206)	38.98** (30.73, 33.61, 50.58)	0.199** (0.197, 0.206),	8.92 (30.84, 37.49, 52.55)
			0.006 (0.005, 0.006)	
*HOUSEHOLD*	0.199*** (0.197, 0.202)	56.69*** (31.57, 35.94, 42.14)	0.199** (0.197, 0.202),	17.85 (29.03, 38.19, 70.31)
			0.091 (0.088, 0.092)	
Panel B: Regression estimates				
Urbanization variable	*RESIDENT*		*HOUSEHOLD*	
	(A)	(B)	(C)	(D)
Threshold variable				
Low regime	0.39**	0.276*	0.291**	0.017
(*TEM*_*it*_≤*φ*_*1*_)	(2.31)	(1.73)	(0.136)	(0.12)
High regime	0.341*	0.226	0.228	-0.042
(*TEM*_*it*_>*φ*_*1*_)	(1.97)	(1.39)	(0.136)	(-0.29)
Independent variables				
*DENSITY*	0.0004	-0.003	-0.001	-0.003
	(0.10)	(-0.83)	(-0.29)	(-0.97)
*GDP*	0.52***	0.605***	0.557***	0.664***
	(10.48)	(9.71)	(15.86)	(11.71)
*HEAVY*	0.099	0.102	0.124	0.136
	(1.24)	(1.34)	(1.3)	(1.61)
*FDI*		-0.012***		-0.013***
		(-5.40)		(-5.25)
*ER*		0.005***		0.005***
		(3.96)		(3.81)
*EC*		0.139**		0.149**
		(2.21)		(2.22)

Notes: Robust standard error is used in regression estimation, *t*-values are shown in parentheses.

"***","**" and "*" denote the significance levels at 1%, 5% and 10%, respectively.

The empirical results suggest that the relationship between urbanization and CO_2_ emissions is non-linear, and that population migration has a marginal effect on the said relationship. During the sample period, migration is found to have weakened the positive impact of resident population urbanization on CO_2_ emissions. The higher the proportion of temporary residents (proxy variable for population migration), the weaker the positive effect of urbanization on CO_2_ emissions. Differences in urbanization stages across regions in China are the main reasons for such results. Usually, regions with a higher proportion of temporary residents tend to have a higher resident population urbanization rate. The main characteristics of a more-developed urbanization stage include stricter environmental supervision by the government, a higher green production capacity, a greater proportion of green consumer products, cleaner energy utilization, and the promotion and development of green transportation industries. By reforming green industry production processes, CO_2_ emissions are reduced. The increase in the proportion of temporary residents increases the availability of labor and improves the human capital through a knowledge spillover effect and labor market competition. This results in an increase in human capital investment, which improves green technology innovation capabilities and strengthened green product supply. Therefore, in regions that are at a higher developed stage of urbanization, an increase in the proportion of temporary residents slow down the rise in CO_2_ emissions.

## 6. Discussion

### 6.1. Explanation from the perspective of population migration

Two findings from our empirical results are intriguing. The first finding is that the excessive agglomeration of urban population plays a greater role than the rural population flow in China’s CO_2_ emissions. One typical feature of China’s population flow is the migration of a large rural population to the cities. The primary factor in the increasing CO_2_ emissions is the excessively agglomerated urban population and the related problems stemming from overcrowding, such as vehicle exhaust emissions and household energy consumption. The rapidly growing urban resident population is accompanied by a rapidly growing demand for motor vehicles, which leads to increased CO_2_ emissions. Moreover, the rapid increase in the urban resident population promotes a large demand for household and commercial energy consumption, further increasing CO_2_ emissions. These results imply that the increase in CO_2_ emissions is closely related to transportation, household energy consumption and commercial energy consumption. However, because the urban resident population will continue to increase, these driving factors will persist unless Chinese cities implement policies to improve energy consumption efficiency and to control CO_2_ emissions. Threshold regression results indicate that the positive relationship between urban resident population and CO_2_ emissions would gradually weaken along with the increase of temporary residents, that is to say, the marginal impact of urban resident population on CO_2_ emissions would gradually decrease.

The second finding is that the urbanization of household registration population will not lead to increased CO_2_ emissions in China. This result seems incredible due to its deviation from the positive correlation between an organized resident population and CO_2_ emissions. Although the population can migrate freely between cities and rural areas, it is difficult for rural migrants to change their permanent residence due to the restrictions imposed by the hukou system. That policy is frankly unfair to rural migrants because the allocation of education, medical treatment and housing are contingent on urban household registration. Generally speaking, persons who can obtain access to urban household registration, especially in large cities, must demonstrate some type of outstanding performance, such as a good educational background, excellent professional skills or successful entrepreneurship. In contrast, this part of the urban household registration population has a more urgent desire and a better ability to improve urban environmental quality through either technological innovation or environmental regulations. Meanwhile, the household registration policy prevents excessive population migration to the large cities, which potentially results in a more reasonable population arrangement nationwide and reduces the pressure of CO_2_ emissions in large cities. However, a long-acting mechanism should be implemented to maintain reasonable population migration instead of relying on the mandatory constraints of the hukou system.

### 6.2. Comparing the ecological modernization and urban environmental transition theories

According to Poumanyvong and Kaneko [7], the ecological modernization theory emphasizes that urbanization is a process of social transformation. Environmental problems may rise from the initial to the middle stages of development due to prioritization of economic growth over environmental sustainability. However, environmental damage may be minimized during the advanced stages of development through technological progress and the shift towards a service-based economy. The urban environmental transition theory details different types of environmental problems and their evolution at different stages of urban development. It argues that cities often become wealthier and in that event, pollution-related (water and air pollution) and consumption-related (energy consumption and CO_2_ emissions) issues become more prominent. These issues can be decoupled via technological progress or other measures.

A comparison of our results on the relationship between urbanization and CO_2_ emissions to these two theories is thought provoking. This paper analyzes the effects of urbanization on CO_2_ emissions from the standpoint of population migration. The relationship between urbanization of resident population and CO_2_ emissions is significantly positive, which is the complete opposite of the relationship between urbanization of household registration population and CO_2_ emissions. Threshold regression results indicate that the marginal impact of urban resident population on CO_2_ emissions would gradually decrease. There is a potential similarity between our results and these two theories, in that our results potentially reflect the effects of urbanization on CO_2_ emissions at different stages of urban development, i.e., the positive correlation between urbanization of resident population and CO_2_ emissions corresponds to the increasing environmental problems at the early stages of development, whereas the negative correlation between urbanization of household registration population and CO_2_ emissions corresponds to the decreasing environmental problems at the later stages of development. Considering China’s circumstances related to urban development during the sample period, CO_2_ emissions show an upward trend in the early stages of urban development because of excessive population aggregation and industrial development, which involves high levels of both pollution and energy consumption. However, this trend declines in the more advanced stages of urban development because society comes to realize the significance of environmental sustainability and has the ability to solve environmental problems through both technological innovation and transformation of the industrial structure.

## 7. Conclusions and policy implications

Using panel data from thirty provinces (municipalities and autonomous regions) in China from 2000 to 2012, and applying the STIRPAT model, this study empirically investigates the impact of urbanization on CO_2_ emissions. Considering the unique characteristics of China’s urban population structure and inter-provincial migration, this study introduces two urbanization indicators to measure the impact of urbanization on CO_2_ emissions. In addition, the proportion of temporary residents across regions is introduced as a proxy variable to empirically explore the marginal impact of population migration on the relationship between urbanization and CO_2_ emissions. The results show that the impact of urbanization on CO_2_ emissions is closely correlated to urban population structure. The proportion of temporary residents reduces the positive effect of resident population urbanization on CO_2_ emissions. It is found that resident population urbanization has a significant positive effect on CO_2_ emissions, while the effect of household registration population urbanization on CO_2_ emissions is significantly negative. The estimation results of the panel threshold model reveal that as the proportion of temporary residents increases, the positive effect of resident population urbanization on CO_2_ emissions becomes weaker. Differences in urbanization stages across regions are the main reason for the generation of such a marginal effect. The following policy suggestions are proposed based on the findings of this study:

1) The innovation pattern of urban governance. According to the conditions of urban population, economic development, industrial structure and resource endowment, it is necessary to perfect city classification and function orientation of different provinces (municipalities and autonomous regions), improve the industrial investment efficiency of different cities, and optimize the industrial distribution of different cities. Cities in other regions can learn from the pollution prevention and control mechanisms of cities in highly developed urbanization areas as well as the construction experience of green development zones. It is also necessary to smooth the channels of public participation to urban environmental pollution monitoring system, and build multi-field environmental risk alerting and emergency response systems. In order to lower the correction cost of deviation from the ecological and social benefit objectives, we ought to strengthen the frontier research of urban green construction projects. These methods are not only beneficial for green urban development in China, but also of reference value for other developing economies, such as Southeast Asian nations and India.

2) The implementation of a differentiated urbanization strategy. Differences in the urbanization stages of provinces (municipalities and autonomous regions) is an objective law of urbanization. In regions with higher rates of urbanization, the government should adopt a stronger guiding role towards green development, cleaner enterprise productivity, the accumulation of high-quality human capital from the external labor market, and the initiatives of green consumption lifestyles. Regions with lower urbanization rates should focus on the coordination and unification of industry and green development. In such cases, more attention should be paid to ecological and environmental protection while pursuing economic development. These measures can effectively control increases in total CO_2_ emissions.

3) The optimization and adjustment of urban population development policy. The urban population lies at the core of high-quality urbanization. The migrating population is an important component of urbanization that should be justly considered in the development of urban population policy. Cities in different regions should aim to formulate effective population policies that address their unique development stages, and to accelerate the citizenization of the population transferred from the agricultural sector, while formulating talent acquisition policies according to regional needs and improving basic public services.

## Supporting information

S1 File Data of regression estimation. (DTA)

S2 File (DO)

S1 Fig Data of Fig 1. (XLSX)

S2 Fig Data of Fig 2. (XLS)
